# Can the difference in medical fees for self and donor freeze-thaw embryo transfer cycle, be in fact a cover-up for the sale of donated human embryos?

**DOI:** 10.1186/1747-5341-2-3

**Published:** 2007-03-29

**Authors:** Boon Chin Heng

**Affiliations:** 1 National University of Singapore, 5 Lower Kent Ridge Road, 119074 Singapore

## Commentary

In recent years, clinical assisted reproduction techniques (ART) are increasingly being practiced worldwide, which in turn has led to an accumulated surplus of cryopreserved embryos within fertility clinics [1,2]. Nevertheless, in those countries where the commercialization of embryo donation is banned – and former ART patients are expected to donate their cryopreserved supernumerary embryos altruistically to infertile couples – the resultant effect is often severe shortages and long waiting lists for prospective recipients [3]. Couples who have attained success in clinical assisted reproduction are usually reluctant to donate their cryopreserved supernumerary embryos to other couples [4]. This is because frozen embryos are psychologically conceptualized by former patients as biologic tissue, living entities, 'virtual' children having interests that must be considered and protected, siblings of their living children, genetic or psychological 'insurance policies', and symbolic reminders of their past infertility [5]. Under such circumstances, whereby legally donated embryos are in such short supply and high demand, medical professionals and health institutions can easily exploit the situation for profiteering in medical fees, particularly if they possess exclusive rights of distribution of the donated embryos to prospective recipients.

Although such institutions are not permitted to directly profit from the transaction of cryopreserved embryos between donor and recipient, it must be remembered that there is still considerable opportunity for profiteering in medical fees arising from laboratory and clinical services rendered to the recipient. It is easy to disguise the 'sale' of altruistically donated human embryos through substantially increased medical fees, particularly in a private practice setting. Firstly, patients seeking treatment at privately run IVF programs are already prepared to pay higher medical fees for the supposedly 'better' reputation of the fertility doctor and 'enhanced' medical facilities available. Secondly, it is often the case that patients seeking donated embryos at a privately-run fertility clinic have already been exasperated by the long waiting list of government-funded IVF programs, and therefore would have no qualms with paying higher medical fees. Lastly, a privately run IVF program would usually treat the medical fees charged to individual patients as confidential information. Hence, it is difficult for patients to discern whether there is any disparity in medical fees for a self- and donor-freeze/thaw embryo transfer cycle, which could, in fact, mask 'opportunistic profiteering' by medical professionals.

Therefore, the pertinent question that arises is, what would constitute a fair and honest profit margin for the medical professional and health institution in question? A suitable benchmark would obviously be the level of medical fees normally charged to patients for a self-freeze/thaw embryo transfer cycle, after initial failure at their first attempt in ART. This is because the level of medical expertise, clinical and laboratory services required for a donor- and self-freeze/thaw embryo transfer cycle should, in theory, be about the same [6], although slight variation in treatment can be expected owing to patients' respective medical histories, which results in varying predisposition to medical complications. In any case, the health authority should ensure that there is no gross disparity in the medical fees for both donor- and self-freeze/thaw embryo transfer cycle, as this could mask opportunistic profiteering by medical professionals and, in fact, be a covert form of embryo commercialization.

There is nothing deplorable about profit-making in healthcare services – after all, medical professionals ought to make a decent living after numerous years of expensive education, coupled with their heavy investment in medical equipment and facilities. Nevertheless, it is imperative that they should always work towards a fair and honest profit margin, rather than exploit the short supply of legally donated human material for financial gain. It must always be remembered that the embryo donor has presumably been altruistically, rather than financially, motivated to help the recipient; such opportunistic profiteering by medical professionals would be an extreme breach of medical ethics and professional code of conduct, particularly if they are encouraging their former patients to altruistically donate supernumerary embryos for the sake of infertile couples. It must be noted that many childless couples feel a sense of exhilaration and euphoric joy upon attaining success in clinical assisted reproduction. This altered emotional state could render them particularly vulnerable to the coaxing of medical professionals, to whom they feel they owe a debt of gratitude [7].

Another solution for greater transparency would be to establish a government-controlled national or regional register of prospective embryo donors and recipients, one successful example being CECOS in France [8]. This would then facilitate a more equitable distribution of donated embryos to prospective recipients based on medical condition and needs, rather than choice of medical doctor and health institution for treatment, the latter providing much room for abuse and opportunistic profiteering.
